# Melanogenesis and Antityrosinase Activity of Selected South African Plants

**DOI:** 10.1155/2012/374017

**Published:** 2012-04-24

**Authors:** Manyatja Brenda Mapunya, Roumiana Vassileva Nikolova, Namrita Lall

**Affiliations:** ^1^Department of Biodiversity, School of Molecular and Life Sciences, University of Limpopo (UL), Private Bag X1106, Sovenga 0727, South Africa; ^2^Department of Plant Science, University of Pretoria, Pretoria 0002, South Africa

## Abstract

Melanin is the pigment that is responsible for the colour of eyes, hair, and skin in humans. Tyrosinase is known to be the key enzyme in melanin biosynthesis. Overactivity of this enzyme leads to dermatological disorders such as age spots, melanoma and sites of actinic damage. Ten plants belonging to four families (Asphodelaceae, Anacardiaceae, Oleaceae, and Rutaceae) were investigated for their effect on tyrosinase using both L-tyrosine and L-DOPA as substrates. Ethanol leaf extracts (500 *μ*g/mL) of *Aloe ferox, Aloe aculeata, Aloe pretoriensis*, and *Aloe sessiliflora* showed 60%, 31%, 17%, and 13% inhibition of tyrosinase activity respectively, when L-tyrosine was used as a substrate. *Harpephyllum caffrum* (leaves) at a concentration of 500 *μ*g/mL had an inhibitory effect of 70% on tyrosinase when L-DOPA was used as a substrate. The IC_50_ of *Harpephyllum caffrum* (leaves and bark) were found to be 51 ± 0.002 and 40 ± 0.035 *μ*g/mL, respectively. Following the results obtained from the tyrosinase assay, extracts from *Harpephyllum caffrum* were selected for further testing on their effect on melanin production and their cytotoxicity on melanocytes *in vitro*. The IC_50_ of both extracts was found to be 6.25 *μ*g/mL for melanocyte cells. Bark extract of *Harpephyllum caffrum* showed 26% reduction in melanin content of melanocyte cells at a concentration of 6.25 *μ*g/mL. The leaf extract of this plant showed some toxicity on melanocyte cells. Therefore, the bark extract of *Harpephyllum caffrum* could be considered as an antityrosinase agent for dermatological disorders such as age spots and melasoma.

## 1. Introduction

Melanin is a pigment that occurs in humans, fungi, and plants [1]. It is responsible for the colour of eyes, hair, and skin in humans [2]. The pigment is secreted and produced by the melanocytes cells, which are distributed in the basal layer of the dermis, through a physiological process called melanogenesis [2–5]. It is formed through a series of oxidative reactions involving the amino acid tyrosine in the presence of the enzyme tyrosinase. There are two types of melanin pigments that can be produced by melanocyte cells, namely, eumelanin which is black or brown, and pheomelanin which is red or yellow and alkali soluble [6, 7]. The colour of human hair and skin is determined by the type or distribution and degree of melanin pigment. Each individual of different racial group has more or less the same number of melanocyte cells, thus the type of melanin produced depends on the functioning of the melanocytes, for example, people with darker skin are genetically programmed to constantly produce higher levels of melanin [2, 6, 7]. The major structural differences between dark and light skins in terms of pigmentation are melanosome (organelles within the melanocyte cells) size and grouping. Melanosomes are smaller and grouped in clumps in light skin, while they are larger single organelle in dark skin [7, 8]. The role of melanin is to protect the skin against UV light damage by absorbing UV sunlight and removing reactive oxygen species [2, 3, 7].

The key enzyme that is responsible for melanin production is tyrosinase [9]. Hyperpigmentation of the skin occurs due to overactivity of tyrosinase enzyme and its underactivity leads to hypopigmentation of hair. Overactivity of the enzyme is associated with ageing while under-activity can occur in any age group depending on a person's heredity [10]. Tyrosinase, also known as polyphenol oxidase, is a copper containing monooxygenase that catalyzes two distinct reactions involving molecular oxygen: hydroxylation of tyrosine to 3,4-dihydroxyphenylalanine (DOPA) by monophenolase action and oxidation of DOPA to DOPA-quinone by diphenolase action [9, 11]. Quinones are highly reactive compounds and can polymerize spontaneously to form high-molecular-weight compounds or brown pigments [12]. Apart from animals, tyrosinase is also widely distributed in plants and is a very important enzyme in controlling the quality of fruits and vegetables. It catalyzes the oxidation of phenolic compounds to the corresponding quinones and is responsible for the enzymatic browning of fruits and vegetables, which are of economic importance [2].

There is a variety of plants that are used traditionally for the treatment of different skin problems. Poor skin penetrations and mutagenic effects of chemically derived compounds such as hydroquinone [9] used in cosmetics led to the search for alternative herbal and pharmaceutical agents to treat skin hyperpigmentation. Aloe species were selected in this study because they are related species to *Aloe vera* which is used in the markets for depigmentation purposes [13]. Other plants, selected in the present study (Table 1), are traditionally used in South Africa for skin-lightening purposes and/or for removing marks or pigments on the face [14]. Different parts of these plants are ground and used as facial masks to remove spots and they are also used for skin lightening purposes. The aim of this study was to test the effect of the selected plant extracts on tyrosinase enzyme and to identify which plant extracts can be used as possible skin-lightening agents.

## 2. Materials and Methods

### 2.1. Plant Material Collection and Extraction

Leaves and bark of selected plant species (Table 1) were collected from the Manie van der Schijff Botanical Garden of the University of Pretoria in July 2006. Some plant materials (*Harpephyllum caffrum* and *Calodendrum capensis* both leaves and bark) were dried in shade while leaves of *Aloe* species were used fresh. Traditionally paste of plant material mixed with water is used for removing hyperpigmentation. However, due to ethanol being a comparatively safer solvent (according to polarity) and its polarity being not very different from that of water, and due to its antiseptic nature, this solvent was chosen for the preparation of extracts. Forty grams of each plant material was ground with 200 mL absolute ethanol using a Jannke & Kunkel grinder. Mixtures were left overnight and then filtered through a Whatman filter paper (15 cm). The solvent was removed under a vacuum (BUCHI, Rotavapor, R-200) to yield dry extracts.

### 2.2. Chemicals and Reagents

Mushroom tyrosinase with the activity of 6680 units/mg and Kojic acid (positive control) were purchased from Sigma-Aldrich. Fetal calf serum (FCS), trypsin, EDTA, L-glutamine, potassium phosphate buffer (pH 6.5), penicillin/streptomycin/fungizone, and sodium pyruvate were purchased from Highveld Biological. The Cell Proliferation Kit II (XTT) (sodium 3-[1-(phenylaminocarbonyl), 4-tetrazolium]-bis (4-methoxy-6-nitro) benzene sulfonic acid hydrate) labeling reagent) was purchased from Roche Diagnostics.

### 2.3. Tyrosinase Enzyme Assay

The assay was performed using relevant methods [9, 15]. Each powdered plant extract was dissolved in dimethyl sulphoxide (DMSO) to a final concentration of 20 mg/mL. This extract stock solution was then diluted to 600 *μ*g/mL in 50 mM potassium phosphate buffer (pH 6.5). Serial dilutions were made to get eight concentrations. Kojic acid was used as a control drug. In a 96-well plate, 70 *μ*L of each extract serial dilution was combined with 30 *μ*L of tyrosinase (333 Units/mL in phosphate buffer) in triplicates. After incubation at room temperature for 5 minutes, 110 *μ*L of substrate (2 mM L-tyrosine or L-DOPA) was added to each well. Final concentrations of the extract samples ranged from 3.91 to 500 *μ*g/mL. The final percentage of DMSO was 1% after the dilution. Optical densities of the reaction mixtures in the wells were then recorded at 492 nm with the BIO-TEK Power Wave XS multi-well plate reader. Final concentration of Kojic acid ranged from 3.125 *μ*g/mL to 400 *μ*g/mL. Plant extracts which showed good antityrosinase activity at a concentration of 60 *μ*g/mL were further investigated for their effect on melanin synthesis by melanocyte cells.

### 2.4. Melanocyte Cell Culture for the Investigation of Melanin Inhibition by Plant Extracts

#### 2.4.1. Preparation of Melanocyte Cell Culture

Mouse melanocyte cell line, B16-F10, was cultured in Dulbecco's Modified Eagle's Medium (DMEM) containing 10% fetal bovine serum, 1.5 g/L NaHCO_3_, 2 mM L-glutamine, 10 *μ*g/mL penicillin, 10 *μ*g/mL streptomycin, and 0.25 *μ*g/mL fungizone and incubated at 37°C with 5% CO_2_ in a humidified atmosphere. Cells were subcultured in a ratio of 1 : 3 on every third or fourth day.

A cell suspension of 1 × 10^5^ B16-F10 cells was prepared in complete DMEM, supplemented with 10% FCS, and (10 mL) antibiotics (penicillin/streptomycin/fungizone). On day 0, B16-F10 cells in complete DMEM were dispensed into the wells of a 96-well plate (10^5^ cells per well) and 24-well plate (10^4^ cells per well). After an overnight incubation at 37°C in 5% CO_2_ and a humidified atmosphere, extract samples were added to the cells to the final concentration of 500, 250, 125, 62.5, 31.25, 15.62, 7.81, and 3.91 *μ*g/mL. Kojic acid was used as a control drug. Final concentration of Kojic acid ranged from 400 to 3.125 *μ*g/mL. Incubation at 37°C in 5% CO_2_ and a humidified atmosphere followed for three days.

#### 2.4.2. Effect of Plant Extracts on Melanin Synthesis

The effect of the plant extracts on melanin synthesis was determined by washing the melanocyte cells in the 24-well plate with potassium phosphate buffered saline (PBS), and lysing with 200 *μ*L of sterile distilled water. Optical densities were recorded at a wavelength of 405 nm. The effect of extracts on melanin production was determined by comparing to the control sample (medium with DMSO).

#### 2.4.3. Toxicity Effect of Plant Extracts

The toxicity of the extracts on the B16-F10 cells was tested using the XTT cytotoxicity assay. Fifty microliters of XTT reagent (1 mg/mL XTT with 0.383 mg/mL PMS) was added to the wells and incubated for one hour. The optical densities of the wells were then measured at 450 nm (690 nm reference wavelength). By comparing to the control (DMEM with DMSO), cell survival was assessed.

### 2.5. Statistical Analysis

The results were analysed statistically using one-way analysis of variance (ANOVA) and the least significant differences (*P* < 0.01) were determined according to Duncan's *t*-test.

## 3. Results

### 3.1. Effect of Plant Extracts on Tyrosinase Activity

Ethanol extracts from different parts of ten selected plants (Table 1) and Kojic acid (positive control) differed in their inhibitory effect on tyrosinase activity when using both L-tyrosine and L-DOPA as substrates. Extracts from *A. arborescens *(leaves), *A. vera* (leaves), *C. capensis* (bark and leaves), and nut oil extract of *S. birrea* and* X. americana* did not inhibit the tyrosinase activity at tested concentrations (3.91 to 500 *μ*g/mL), see Table 2. However, at a concentration of 500 *μ*g/mL the leaf extracts of *A. aculeata*,* A. pretoriensis* and *A. sessiliflora* showed 31%, 17%, and 13% inhibition of tyrosinase enzyme, respectively (Table 2). Leaf extract from *Aloe ferox* showed inhibition of tyrosinase by 60%, 51% and 48% at 500 *μ*g/mL, 250 *μ*g/mL, and 125 *μ*g/mL, respectively. Leaf and bark extracts of *H. caffrum* showed significant (*P* < 0.01) inhibition of the enzyme by 90% and 92% at 500 *μ*g/mL, respectively, as compared to all other extracts tested (Table 2).

Plant extracts which exhibited inhibition of tyrosinase at 500 *μ*g/mL when using L-tyrosine as a substrate were further tested for their effect on tyrosinase activity using L-DOPA as a substrate. Plants extract tested were *H. caffrum* (bark and leaves), *A. aculeata*, *A. ferox*, *A. pretoriensis, *and *A. sessiliflora* (leaves). *A. pretoriensis* and *A. sessiliflora* leaf extracts did not show any inhibition of tyrosinase even at the highest concentration tested (Table 2). Extracts of *H. caffrum* (bark and leaves) had stronger inhibitory effect on tyrosinase than the other plant extracts tested for tyrosinase activity using L-DOPA as a substrate (Table 2). Kojic acid also had strong inhibitory effect on tyrosinase (88%, 83%, 74%, and 63% at concentrations of 400, 200, 100, and 50 *μ*g/mL, resp.) when L-DOPA was used as a substrate. The IC_50_ of *Harpephyllum caffrum* (leaves and bark) were found to be 51 ± 0.002 and 40 ± 0.035 *μ*g/mL, respectively (Table 3).

### 3.2. Effect of Plant Extracts on Melanin Biosynthesis by Mouse Melanocytes

**Figure 1:**
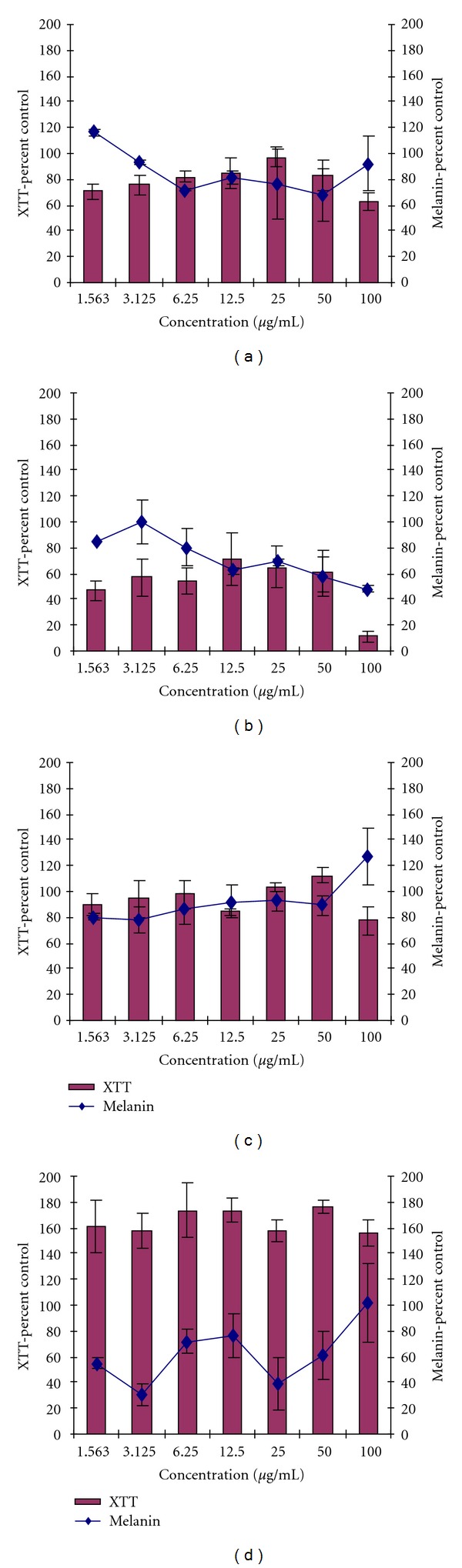
The effect of samples on cell viability/proliferation and melanin production by melanocyte cells, *H. caffrum* bark (a), leaves (b), *A. arborescence* leaves (c), and Kojic acid (d).

Following the results obtained from tyrosinase assay, extracts from *H. caffrum* (leaves and bark) were selected for further testing on their effect on melanin production and their cytotoxicity on melanocytes *in vitro* since they had an inhibitory effect on tyrosinase when using both L-tyrosine and L-DOPA as substrates. The leaf extract from *Aloe arborescens* was selected to test its potential to promote melanin production since it had no inhibitory effect on the tyrosinase. The bark extract of *H. caffrum* showed 26% reduction in melanin content of melanocyte cells at a concentration of 6.25 *μ*g/mL (Figure 1(a)) while the leaf extract of *A. arborescens* showed 23% reduction in melanin content at the same concentration. The leaf extract of *H. caffrum* showed toxicity to melanocyte cells at most concentrations tested (Figure 1(b)).

### 3.3. Toxicity Effect of Plant Extracts

Extracts from *H. caffrum* (bark) and *A. arborescens *both showed low toxicity effect on melanocyte cells (Figures 1(a) and 1(c)) at all concentrations tested with cell viability above 80%, and 70%. However, leaf extracts of *H. caffrum* showed toxicity to melanocytes cells (Figure 1(b)) at a concentration of 100 *μ*g/mL. Kojic acid showed reduction in melanin production by melanocyte cells with 69% and 61% at 3.12 *μ*g/mL and 25.0 *μ*g/mL, respectively (Figure 1(d)), and was not toxic to melanocyte cells at all concentrations tested with cell viability of above 80%.

## 4. Discussion

It is reported that *Aloe vera*'s derived compounds are used in skin-lightening agents [16] while from our results *Aloe vera* did not show any inhibition of tyrosinase. Active compounds against tyrosinase from *Aloe vera* were isolated from the sap of the leaves [16, 17]. The aloes used in this study lacked the sap which may be due to the seasonal variation, even though the time and season of plant's collection in other studies was not specified.

*Aloe arborescens* had no inhibitory activity on tyrosinase but when tested on melanocyte cells showed a reduction of melanin production by 23% instead of an increase. This can be due to the fact that melanin biosynthesis is a multi- step pathway [1, 10] and thus the extracts may act on other enzymes in the pathway rather than directly on tyrosinase. Leaf extracts of *Aloe pretoriensis* and *Aloe sessiliflora* had an inhibitory effect on tyrosinase when L-tyrosine was used as a substrate but they did not show any activity on tyrosinase when L-DOPA was used as a substrate. This shows that they may act on monophenolase activity of tyrosinase by inhibiting conversion of tyrosine to L-DOPA [9, 11, 15].

The bark extract of *Harpephyllum caffrum* had the highest inhibitory effect on tyrosinase and the highest reduction of melanin production by melanocytes cells as compared to all other plant extracts tested, except for Kojic acid. This extract and the Kojic acid were not toxic on melanocytes cells as compared to the leaf extract of *Harpephyllum caffrum*. The results from this study show that *Harpephyllum caffrum* (bark) has the potential to serve as the source of chemical constituents for antpigmentation treatments.

Kojic acid (positive control) had the highest inhibitory effect on tyrosinase as compared to extracts of *Harpephyllum caffrum*. It also resulted in a higher reduction of melanin production by melanocyte cells (38% at 6.25 *μ*g/mL) as compared to the bark extract of *Harpephyllum caffrum* (26% at 6.25 *μ*g/mL). Kojic acid is reported to cause skin irritation when applied topically [18] but did not show any toxicity on the melanocyte cells in concentrations tested (400 *μ*g/mL to 3.125 *μ*g/mL) in this study.

## 5. Conclusions

From the present study, it can be concluded that scientific validation of the plant extracts used traditionally for treatment of skin, age-spots, dark marks, skin-lightening, and so forth is necessary in order to investigate their potential as skin-lightening agents. Bark extract of *Harpephyllum caffrum,* which exhibited good antityrosinase activity, inhibited melanin production in cell cultures, and did not show a toxic effect, will require further investigations in clinical studies in order to determine its potential as a tyrosinase inhibitor.

## Figures and Tables

**Table 1:** List of selected plants and their traditional uses.

Plants	Common names	Family name	Medicinal use
*Aloe aculeata* Pole-Evans (Leaves)	Ngopane	Asphodelaceae	Used as a skin lightening [19]
*Aloe arborescens *Mill. (Leaves)	Ikalane/Umhlabana	Asphodelaceae	Leaf extracts have shown to have significant wound healing, antimicrobial, anti-ulcer and anticarcinogenic activity [14]
*Aloe ferox* Mill. (Leaves)	Ikhala/Inhlaba	Asphodelaceae	Sap in the leaves used traditionally as laxatives and can be taken for arthritis [14]
*Aloe pretoriensis* Pole-Evans (Leaves)	N/A	Asphodelaceae	Used as a skin lightening [19]
*Aloe sessiliflora* Pole-Evans (Leaves)	N/A	Asphodelaceae	Used traditionally to treat the uterus and believed to promote menstruation [14]
*Aloe vera* (L.) Burm.f. (Leaves)	N/A	Asphodelaceae	The gel from leaves is used as a remedy for minor burns and scrapes and for sunburn [14]
*Calodendrum capensis *Thumb. (Leaves)	Umbhaba	Rutaceae	Used as a facial mask [19]
*Calodendrum capensis* Thumb. (Bark)	Umbhaba	Rutaceae	Used traditionally in soaps and as a skin-lightener as white umemezi [14]
*Harpephyllum caffrum *Bernh. (Leaves)	Umgwenya	Anacardiaceae	Used as a face mask [19]
*Harpephyllum caffrum *Bernh. (Bark)	Umgwenya	Anacardiaceae	Acne and eczema treatment, and is usually applied as facial saunas and skin washes [14]
*Sclerocarya birrea *(A. Rich.) Hochst. (Nuts)	Morula	Anacardiaceae	Oil extracted from the kernels is Africa's greatest skin care oil and as a skin-lightener (personal communication) [14]
*Ximenia americana* L. (Nuts)	Umthunduluka-obmvu	Olacaceae	Seeds contain valuable oil that is used traditionally to soothe leather and as cosmetic and skin ointment [14]

*N/A Not available.*

**Table 2:** Inhibitory activity of selected plants on tyrosinase when both tyrosine and L-DOPA are used.

Plant extracts [500 *μ*g/mL]	(%)	
	Tyrosine	L-DOPA
*Aloe aculeata* Pole-Evans (Leaves)	31	—
*Aloe arborescens* Mill. (Leaves)	—	*
*Aloe ferox* Mill. (Leaves)	60	—
*Aloe pretoriensis* Pole-Evans (Leaves)	17	—
*Aloe sessiliflora* Pole-Evans (Leaves)	13	—
*Aloe vera* (L.) Burm.f. (Leaves)	—	*
*Calodendrum capensis *Thumb. (Leaves)	—	*
*Calodendrum capensis* Thumb. (Bark)	—	*
*Harpephyllum caffrum *Bernh. (Leaves)	90	70
*Harpephyllum caffrum *Bernh. (Bark)	92	60
*Sclerocarya birrea *(A. Rich.) Hochst. (Nuts)	—	*
*Ximenia americana* L. (Nuts)	—	*

*not tested, —not active.

**Table 3:** The IC_50_ (concentrations at which half the tyrosinase activity is inhibited) values of active plant extracts and positive control.

Plant extracts/ Positive control	IC_50_ (Tyrosine) *μ*g/mL	IC_50_ (L-DOPA) *μ*g/mL
*Harpephyllum caffrum* (Leaves)	51 ± 0.002	125 ± 0.08
*Harpephyllum caffrum* (Bark)	40 ± 0.035	250 ± 0.12
Kojic acid	2.145 ± 0.082	26.66 ± 0.104
